# Treatment with 17β-Estradiol Reduced Body Weight and the Risk of Cardiovascular Disease in a High-Fat Diet-Induced Animal Model of Obesity

**DOI:** 10.3390/ijms18030629

**Published:** 2017-03-14

**Authors:** Wei-Jen Ting, Chih-Yang Huang, Chong-He Jiang, Yueh-Min Lin, Li-Chin Chung, Chia-Yao Shen, Peiying Pai, Kuan-Ho Lin, Vijaya Padma Viswanadha, Shih-Chieh Liao

**Affiliations:** 1 The Sixth Affiliated Hospital of Guangzhou Medical University, Qingyuan People’s Hospital, B24 Yinquan South Road, Qingyuan 511518, China; tim_sigeen@hotmail.com; 2 Graduate Institute of Basic Medical Science, China Medical University, 91 Hsueh-Shih Road, Taichung 40402, Taiwan; cyhuang@mail.cmu.edu.tw; 3 Graduate Institute of Chinese Medical Science, China Medical University, 91 Hsueh-Shih Road, Taichung 40402, Taiwan; 4 Department of Health and Nutrition Biotechnology, Asia University, 500 Lioufeng Road, Taichung 41354, Taiwan; 5 Department of Urology, The Sixth Affiliated Hospital of Guangzhou Medical University, Qingyuan People’s Hospital, B24 Yinquan South Road, Qingyuan 511518, China; 00687@qyry.com; 6 Department of Pathology, Changhua Christian Hospital, 135 Nanxiao Street, Changhua 50006, Taiwan; 93668@cch.org.tw; 7 Department of Medical Technology, Jen-Teh Junior College of Medicine, Nursing and Management, 79-9 Sha-Luen Hu, Hou-Loung Town, Miaoli 35664, Taiwan; 8 Department of Hospital and Health Care Administration, China Nan University of Pharmacy & Science, 60, Section 1, Erren Road, Rende District, Tainan 71710, Taiwan; alanjack@mail.cnu.edu.tw; 9 Department of Nursing, Mei Ho University, 23 Pingguang Road, Pingtung 91202, Taiwan; x00003061@meiho.edu.tw; 10 Division of Cardiology, China Medical University Hospital, 91 Hsueh-Shih Road, Taichung 40402, Taiwan; paidoctor1@gmail.com; 11 Department of Emergency Medicine, China Medical University Hospital, 91 Hsueh-Shih Road, Taichung 40402, Taiwan; cathyshibu@gmail.com; 12 Department of Biotechnology, Bharathiar University, Coimbatore 641046, India; padma.vijaya@gmail.com; 13 School of Medicine, College of Medicine, China Medical University, 91 Hsueh-Shih Road, Taichung 40402, Taiwan

**Keywords:** estrogen receptor α, estrogen receptor β, 17β-estradiol, heart protection

## Abstract

Estrogen receptor α (ERα) and estrogen receptor β (ERβ) play important roles in cardiovascular disease (CVD) prevention. Recently, these estrogen receptors were reconsidered as an important treatment target of obesity leading to CVD. In this study, 17β-estradiol (17β-E) replacement therapy applied to high-fat diet-induced obese C57B male mice and ovariectomized (OVX) rats were evaluated, and the protective effects against high-fat diet-induced obesity were assessed in C57B mouse hearts. The results showed that 17β-E treatment activated both ERα and ERβ, and ERβ levels increased in a dose-dependent manner in high-fat diet C57B mouse cardiomyocytes following 17β-E treatment. Notably, an almost 16% reduction in body weight was observed in the 17β-E-treated (12 μg/kg/day for 60 days) high-fat diet-induced obese C57B male mice. These results suggested that 17β-E supplements may reduce CVD risk due to obesity.

## 1. Introduction

Estrogen receptors α (ERα) and β (ERβ) are two kinds of isomers in humans. Both receptors are found in males and females and have different functions [1]. Many studies have shown that they are closely related to insulin resistance as well as obesity, especially before and after menopause [2]. Because obesity increases the risk of cardiovascular disease (CVD), many studies have examined the use of hormonal therapy in cases of hormone-deficient obesity.

The successful outcome of estrogen replacement therapy has encouraged the use of estrogen for prevention of CVD caused by obesity [3,4,5]. In general, in healthy and young women, the risk of CVD is low and decreases after menopause [6]. Our previous research showed that activation of insulin-like growth factor 1 receptor (IGFR) effectively increased the phosphoinositide 3-kinase (PI3K) and protein kinase B (PKB/Akt) signaling pathway and protected heart cells against apoptosis. This protective mechanism may involve the estrogen receptor signaling pathway [7,8,9,10].

Additionally, when the heart produces increased output or shows pathological hypertrophy, interleukin 6 (IL-6) can be secreted by different types of cells, such as T cells, monocytes, macrophages, fibroblasts, keratinocytes, and endothelial cells [11]. Many reports have shown that these cytokines are important in cardiac hypertrophy and induce a series of signaling pathways. For example, IL-6 has a crucial role in activation of the downstream protein Janus protein tyrosine kinase (JAK) and extracellular signal-regulated kinase 5 (Erk5); these changes lead to downstream zinc finger protein transcription factor (GATA4) phosphorylation [12,13,14,15,16,17].

Experiments in transgenic mice showed that activated T cells were generated by nuclear factor of activated T lymphocyte (NFAT-3) and the GATA4, two specific transcription factors that play important roles in cardiac hypertrophy [18]. When the heart is exposed to external stimuli, calcineurin can cause NFAT-3 dephosphorylation, which allows NFAT-3 to enter the nucleus, while p-GATA4 binding activates cardiomyocyte marker genes, such as atrial natriuretic peptide (ANP), brain natriuretic peptide (BNP), and β-myosin heavy chain (β-MHC), resulting in cardiac hypertrophy [18,19,20,21].

Many flavonoids or phytoestrogens, such as Tanshinone IIA, protect the heart against hypertrophy via the estrogen receptor [22,23]. However, there is no direct evidence showing that estrogen has the same protective effect. In this study, we used a 17β-E intraperitoneal (IP) injection animal model and assessed the effects of hormone therapy. The results suggested that 17β-E can prevent CVD caused by high-fat diet-induced obesity in C57B mice.

## 2. Results and Discussion

**Figure 1:**
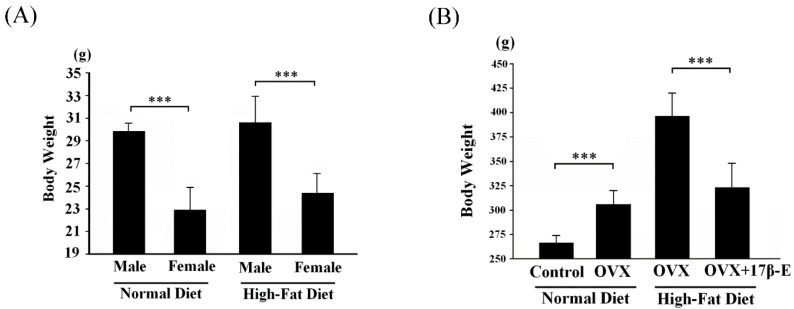
The body weight control effects of estrogen in C57B mice and Sprague-Dawley (SD) rats. (**A**) The body weight of male/female C57B mice fed with normal or high-fat diet for 30 days (*n* = 6 in each group). (**B**) The body weight of female SD rats, ovariectomized (OVX)-female SD rats and high-fat diet-fed OVX-female SD rats with or without 17β-E treatment (12 μg/kg/day) for 30 days. (*** = *p* < 0.001 compared with the indicated group.)

C57B mice were fed a normal diet or a high-fat diet for 30 days, and during this short tern treatment the dietary intake and glucose tolerance of the high fat diet-fed mice were already known present no significantly difference. The average weight of normal diet-fed male C57B mice was 29.8 ± 1.1 g, and the average weight of normal diet-fed female C57B mice was 22.9 ± 2.3 g. The difference between these two normal diet-fed groups was significant (*p* < 0.05) (Figure 1A). The average weight of high-fat diet-fed male C57B mice was 30.8 ± 2.4 g, and the average weight of high-fat diet-fed female C57B mice was 24.2 ± 1.9 g. The difference between these two high-fat diet-fed groups was also significant (*p* < 0.05) (Figure 1A). From the results of the 30-day experiment, male mice had greater weight gain than that of female mice. However, the effect of a high-fat diet on male mice was not significant. 

The average weight of normal diet-fed ovariectomized (OVX) Sprague-Dawley (SD) female rats was 309.1 ± 12.1 g, which was 18% higher than the average weight (262.2 ± 5.7 g) of normal diet-fed SD female rats (control group, sham surgery treatment) within 30 days (Figure 1B). The weight of high-fat diet-fed OVX-SD female rats was 383.6 ± 27.4 g, which was 16% higher than the average weight (321.7 ± 26.9 g) of 17β-E (12 μg/kg/day)-treated high-fat diet-fed OVX-SD female rats within 30 days (Figure 1B). The results showed that endogenous estrogen had a significant effect on body weight in high-fat diet-fed female rats.

**Figure 2:**
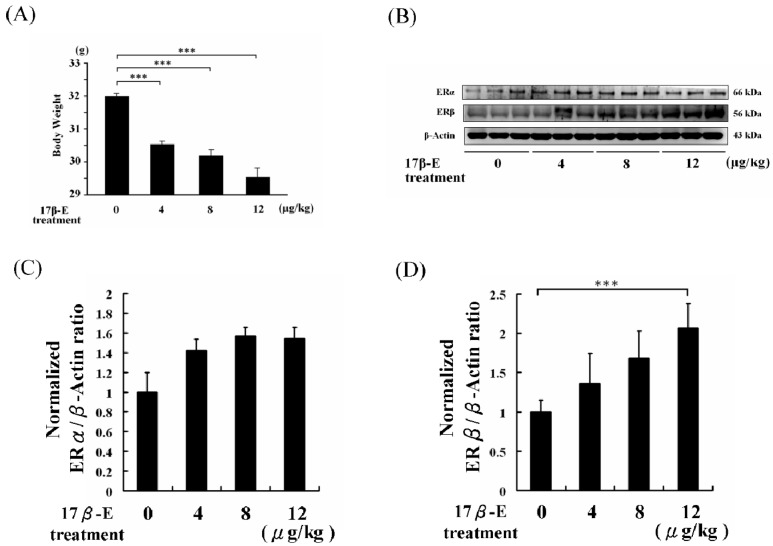
The 17β-E treatment effects on heart estrogen receptors and body weights of 60 days high-fat diet-fed combined with17β-Etreatments (0, 4, 8, and 12 μg/kg/day) male C57B mice (*n* = 6 in each gourp). (**A**) The analysis of body weights. (**B**) ERα and ERβ protein level these high-fat diet-fed male C57B mice heart protein samples. (**C**) The normalized protein expression of ERα/β-actin. (**D**) The normalized protein expression of ERβ/β-actin. (*** = *p* < 0.001 compared with indicated group.)

At 60 days, the average weight of high-fat diet-fed male C57B mice with PBS intraperitoneal (IP) injection (control group) was 32.0 ± 0.2 g (Figure 2A). The average weight of high-fat diet-fed male C57B mice with 17β-E (4 μg/kg/day) IP-injection was 30.6 ± 0.3 g, which was 4.3% lower than that of the control group (Figure 2A). The average weight of the high-fat diet-fed male C57B mice with 17β-E (8 μg/kg/day) IP-injection was 30.3 ± 0.4 g, which was 5.3% lower than that of the control group. The average weight of the high-fat diet-fed male C57B mice with 17β-E (12 μg/kg/day) injection was 29.6 ± 0.5 g, which was 7.5% lower than that of the control (Figure 2A). The physiological character changes of heart weight and gonadal adipose weight are listed in Table 1.

The high-fat diet-fed male C57B mouse hearts were collected, and the ERα and ERβ protein levels were analyzed using Western blotting assays. ERα protein levels were increased in 17β-E-treated high-fat diet-fed male C57B mouse hearts, but the enhancement did not correspond to the doses of 17β-E (Figure 2B). The ERβ protein levels were also increased in 17β-E-treated high-fat diet-fed male C57B mouse hearts in a dose-dependent manner (Figure 2B).

A previous report indicated that 17β-E treatment stimulated ER-α and ER-β expression and reduced appetite and obesity [2]. As shown in this study, 17β-E treatment reduced the body weight of high-fat diet-fed female OVX-SD rats and high-fat diet-fed male C57B mice (Figure 1 and Figure 2). In this work, the concentration of 17β-E (4, 8, and 12 μg/kg/day) may be greater than the endogenous expression in female mice [24,25]. However, the 17β-E IP-injection showed a significant benefit to the high-fat diet-fed male C57B mice with regard to body weight and heart protection. Although the 17β-E treatment showed promising results in this work, not all hormones offer the same benefits. In 2009, Newbold revealed that the supplemental synthetic estrogen diethylstilbestrol (DES) increased obesity in male rats [26].

Furthermore, some CVD risk proteins, such as angiopoietin-like protein 2 (Angpt12) or leptin, secreted from adipose tissues can result in increased inflammation and the development of CVD [27,28]. Several reports have indicated that the expression of ERα, but not ERβ, can increase skeletal muscle lipid oxidation [29]. Recently, Yasrebi revealed that the activation of estrogen response element (ERE)-independent ERα signaling is a possible mechanism by which 17β-E treatment prevents female mice from diet-induced obesity [30]. Unfortunately, the protective mechanism of ER or ERE remains unknown.

**Figure 3:**
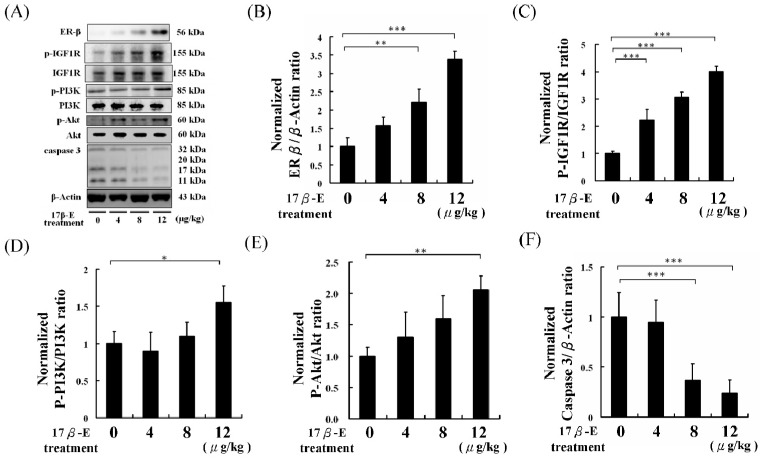
The cardiac survival and apoptosis biomarker analysis of 60 days of different doses of 17β-estradiol treatments (0, 4, 8, 12 μg/kg/day) for high-fat diet-fed male C57B mice (*n* = 6 in each group). (**A**) The hearts protein samples Western blotting analysis results of ERβ, p-IGF1R, IGF1R, p-PI3K, PI3K, p-Akt, Akt, caspase 3, and β-actin. (**B**) The normalized protein expression of ERβ/β-actin. (**C**) The normalized protein expression of p-IGF1R/β-actin. (**D**) The normalized protein expression of p-PI3K/β-actin. (**E**) The normalized protein expression of p-Akt/β-actin. (**F**) The normalized protein expression of caspase 3/β-actin. (* = *p* < 0.05, ** = *p* < 0.01, *** = *p* < 0.001 compared with indicated group.)

However, ERβ seems to be the major estrogen receptor provides protection effects to high-fat diet-induced obese mice hearts [29]. Cardiac survival biomarker proteins, such as p-IGF1R, p-PI3K, and p-Akt, in high-fat diet-fed male C57B mouse hearts were analyzed by Western blotting assays. The results showed that the p-IGF1R, p-PI3K, and p-Akt protein levels were higher in the 17β-E treatment groups than those in the control group (Figure 3).

**Figure 4:**
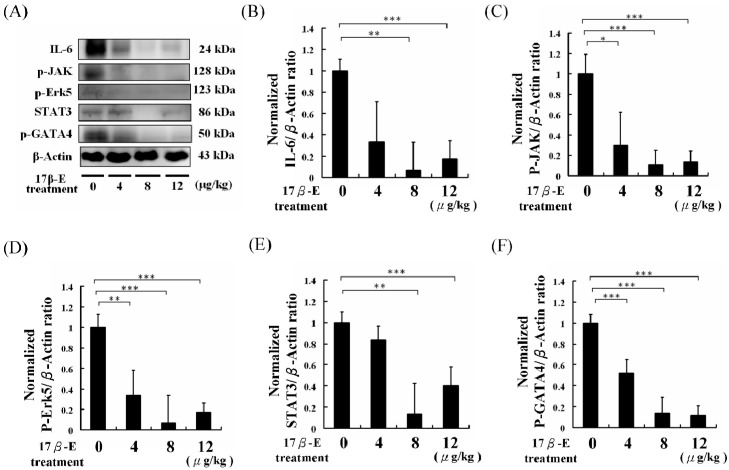
The cardiac hypotrophy and CVD risk biomarker protein analysis of 60 days of different doses of 17β-estradiol treatments (0, 4, 8, 12 μg/kg/day) for high-fat diet-fed male C57B mice heart protein samples. (**A**) The hearts protein samples Western blotting analysis results of IL-6, p-JAK, p-Erk5, STAT3, p-GATA4, and β-actin. (**B**) The normalized protein expression of IL-6/β-actin. (**C**) The normalized protein expression of p-JAK/β-actin. (**D**) The normalized protein expression of p-Erk5/β-actin. (**E**) The normalized protein expression of STAT3/β-actin. (**F**) The normalized protein expression of p-GATA4/β-actin. (* = *p* < 0.05, ** = *p* < 0.01, *** = *p* < 0.001 compared with the control, *n* = 6 in each group.)

Additionally, cardiac apoptosis biomarker proteins, such as caspase 3, were lower in the 17β-E treatment group mice than those of the control group mice (Figure 3). Cardiac hypotrophy and CVD biomarker proteins, such as IL-6, p-JAK, p-Erk5, signal transducer and activator of transcription 3 (STAT3) and p-GATA4, in high-fat diet-fed male C57B mouse hearts were analyzed using Western blotting assays. The p-JAK, p-Erk5, STAT3, and p-GATA4 protein levels were lower in the 17β-E treatments groups than those of the control group (Figure 4).

**Figure 5:**
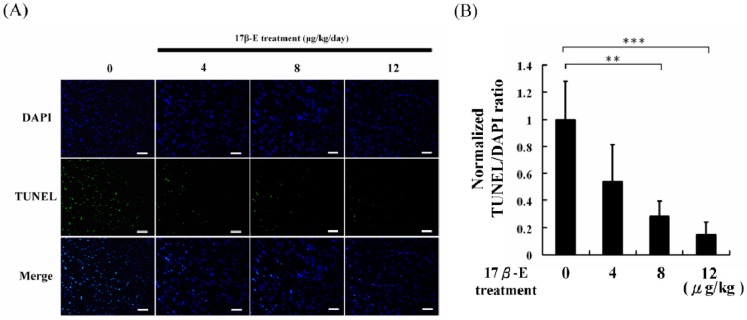
The cardiac apoptosis analysis of the heart slices from 60 days of different doses of 17β-estradiol treatments (0, 4, 8, 12 μg/kg/day) combined with high-fat diet-fed male C57B mice hearts. (**A**) DAPI and TUNEL staining assay were applied to all heart section samples. The nuclei of cells presented in blue color by DAPI stain, and the nuclei of apoptosis cells presented in green color by TUNEL stain (bar length = 50 μm). (**B**) The apoptosis cell ratio in each group slice was presented in normalized TUNEL/DAPI fold. (** = *p* < 0.01, *** = *p* < 0.001 compared with the control, *n* = 6 in each group).

The experimental results of the present study showed that ERβ expression increased in the hearts of high-fat diet-fed male C57B mice in a dose-dependent manner following 17β-E treatments (Figure 2). Indicator proteins of cardiac survival, such as p-IGF1R, p-PI3K, and p-Akt, were also increased along with ERβ expression (Figure 3). Similarly, caspase 3, a cardiac apoptosis marker protein, was also reduced in 17β-E-treated high-fat diet-fed male C57B mouse hearts (Figure 3). The terminal deoxynucleotidyl transferase dUTP nick end labeling (TUNEL) and 4′,6-diamidino-2-phenylindole dihydrochloride (DAPI) dual staining results also confirmed that 17β-E treatments prevented cardiac apoptosis in heart tissue slices (Figure 5). These findings indicate that 17β-E treatments dose-dependently activation of ERβ may directly and the activation of ERα in 17β-E treatments may indirectly protect cardiomyocytes [31,32]. In this work, the results obtained for reasonable explanation to phytoestrogens protection in high-fat diet-fed animal hearts rather than emphasize the application of estrogen in obesity clinical therapy [33].

## 3. Experimental Section

### 3.1. Chemicals

We purchased 17β-estradiol (17β-E, CAS number 50-28-2, empirical formula C_18_H_24_O_2_, molecular weight 272.38) from Sigma-Aldrich (St. Louis, MO, USA).

### 3.2. Animals and Experimental Design

All experimental animals were purchased from the National Laboratory Animal Breeding and Research Center (Taipei, Taiwan), and the experimental protocol was approved by the Institutional Animal Care and Use Committee (IACUC) of China Medical University (No. 102-128-N) on 24 June 2013.

In the initial experiment, 12 male and 12 female C57B mice (5 weeks of age, 20 g weight) were divided into 4 groups (*n* = 6 each). One group consisted of male mice fed a normal diet, one consisted of female mice fed a normal diet, one consisted of male mice fed a high-fat diet, and one consisted of female mice fed a high-fat diet. The experiments lasted for 30 days, and the mice were then sacrificed for further analysis.

In the second set of experiments, 24 female SD rats (4 weeks of age, 200 g weight) were divided into 4 groups (*n* = 6 each). One group consisted of control rats (sham surgery) fed a normal diet, one consisted of OVX-rats fed a normal diet, one consisted of OVX-rats fed a high-fat diet, and one consisted of OVX-rats fed a high-fat diet combined with 17β-EIP-injection (12 μg/kg/day). The OVX surgery was performed 1 week after the rats were purchased because considered a minimal extent of estrogen receptor dependence, and the experiments started 1 week after OVX surgery. The experiments lasted 30 days, and the rats were then sacrificed for further analysis.

In the third set of experiments, 24 male C57B mice (5 weeks of age, 20 g weight) were divided into 4 groups and fed a high-fat diet. One group was administered an IP-injection of PBS, one an IP-injection of 17β-E (4 μg/kg/day), one an IP-injection of 17β-E (8 μg/kg/day), and one an IP-injection of 17β-E (12 μg/kg/day). The treatments were applied for 60 days, and the mice were then sacrificed for further analysis.

All rats and mice were supplied with water ad libitum and housed in standard laboratory conditions at 25 °C, 60% humidity, in a12 h light/dark cycle. The normal diet (Laboratory Rodent Diet 5001) was purchased from LabDiet (St. Louis, MO, USA), and the high-fat diet contained a mixture of 70% (*w*/*w*) normal diet and 30% (*w*/*w*) coconut oil.

### 3.3. Preparation of Protein Extracts from Heart Tissue

The protein samples from heart tissues were harvested in a cold RIPA buffer (1% NP-40, 50 mM Tris base, 0.1% sodium dodecyl sulfate, 0.5% deoxycholic acid, and 150 mM NaCl) containing leupeptin (17 μg/mL) and sodium orthovanadate (10 μg/mL). The heart tissues were homogenized on ice for 5 min. All mixtures were then centrifuged at 14,000 rpm at 4 °C for 15 min, and the protein content of the supernatants was determined with Coomassie blue total protein reagent (Kenlor Industries, Santa Ana, CA, USA) using bovine serum albumin (BSA, 1 mg/mL) as a standard.

### 3.4. Western Blot Analysis

Equivalent amounts of all protein samples (40 μg) were subjected to SDS-polyacrylamide gel electrophoresis and electro transferred to polyvinylidene difluoride (PVDF) membranes (Millipore, Billerica, MA, USA). Membranes were blocked with 3% BSA in Tris-buffered saline (TBS) solution with 0.05% Tween-20 and incubated with primary antibodies, including antibodies against β-actin (sc-47778, Santa Cruz Biotechnology, Dallas, TX, USA), caspase-3 (sc-7148, Santa Cruz Biotechnology, Dallas, TX, USA), ERα (sc-7202, Santa Cruz Biotechnology, Dallas, TX, USA), ERβ (sc-8974, Santa Cruz Biotechnology, Dallas, TX, USA), p-GATA4 (sc-32823-R, Santa Cruz Biotechnology, Dallas, TX, USA), p-Akt (sc-7985, Santa Cruz Biotechnology, Dallas, TX, USA), Akt (sc-5298, Santa Cruz Biotechnology, Dallas, TX, USA), p-PI3K (sc-12929, Santa Cruz Biotechnology, Dallas, TX, USA), PI3K (sc-423, Santa Cruz Biotechnology, Dallas, TX, USA), p-IGF1R (sc-101704, Santa Cruz Biotechnology, Dallas, TX, USA), IGF1R (sc-463, Santa Cruz Biotechnology, Dallas, TX, USA), p-JAK (sc-21870, Santa Cruz Biotechnology, Dallas, TX, USA), p-Erk5 (sc-1284, Santa Cruz Biotechnology, Dallas, TX, USA), IL-6 (sc-7290, Santa Cruz Biotechnology, Dallas, TX, USA), and STAT3 (sc-483, Santa Cruz Biotechnology, Dallas, TX, USA), at 4 °C overnight. The PVDF membranes were then washed three times with TBS solution containing 0.05% Tween-20 and incubated with the secondary antibody at 4 °C for 1 h. Bands were detected by enhanced chemiluminescence using ECL Western blotting detection reagents and exposed using ECL Hyperfilm (FUJFILM Las-4000, Tokyo, Japan). Protein quantity was determined by densitometry using FUJIFILM Multi Gauge (FUJFILM, Tokyo, Japan), version 2.2, software.

### 3.5. Apoptosis Analysis

Cardiac apoptosis of all high-fat diet-fed C57B mouse heart tissue slices was assessed using DAPI and TUNEL staining assays. In brief, sections were incubated with proteinase K for 20 min and then incubated with blocking buffer after two washes. The TUNEL reagent (apoptosis detection kit, Roche Applied Science, Basel, Switzerland) was applied to the slices for 60 min at 37 °C. Apoptosis-positive nuclei (fragmented DNA) showed bright green fluorescence by TUNEL staining and were measured at a wavelength of 460 nm. DAPI PBS solution (0.1 μg/mL) was added to the slides for 5 min, and nuclei showing blue fluorescence were assessed at a wavelength of 454 nm. Photomicrographs were obtained using microscopes (Zeiss Axiophot, Oberkochen, Deutschland, Germany).

### 3.6. Statistical Analysis

All data were analyzed from three independent experiments and are represented as the mean ± standard deviation. Statistical analysis was performed using a one-way analysis of variance (ANOVA) for groups and a Student’s *t*-test for paired samples. *p* < 0.05 was considered statistically significant.

## 4. Conclusions

These experimental evidences showed that 17β-E treatments decreased obesity-induced cardiac hypertrophy and downstream indicator proteins are regulated by estrogen receptor expression in high fat diet-induced obese C57B mice.

## Figures and Tables

**Table 1:** The physiological characters of high-fat diet-fed male C57B mice with different 17β-E treatments. HW = heart weight, TL = tibia length, GAW = Gonadal adipose weight (* = *p* < 0.05; ** = *p* < 0.01).

17β-E (μg/kg/day)	0	4	8	12
HW (g)	0.22 ± 0.03	0.19 ± 0.04	0.16 ± 0.01	0.16 ± 0.03
TL (mm)	22.80 ± 0.13	22.73 ± 0.14	22.59 ± 0.15	22.87 ± 0.12
HW/TL ratio (×10^3^)	9.65 ± 1.31	8.36 ± 1.76	7.08 ± 0.44 **	7.00 ± 1.31 *
GAW (g)	0.25 ± 0.07	0.21 ± 0.03	0.18 ± 0.02	0.17 ± 0.06
